# Orientation-Independent Yield Stress and Activation Volume of Dislocation Nucleation in LiTaO_3_ Single Crystal by Nanoindentation

**DOI:** 10.3390/ma12172799

**Published:** 2019-08-30

**Authors:** Yi Ma, Xianwei Huang, Yuxuan Song, Wei Hang, Julong Yuan, Taihua Zhang

**Affiliations:** 1 College of Mechanical Engineering, Zhejiang University of Technology, Hangzhou 310014, China; 2 Key Laboratory of Special Purpose Equipment and Advanced Manufacturing Technology Ministry of Education, Zhejiang University of Technology, Hangzhou 310027, China; 3 Institute of Solid Mechanics, Beihang University, Beijing 100191, China

**Keywords:** Lithium tantalate, nanoindentation, pop-in, yield stress, orientation effect, activation volume

## Abstract

Relying on nanoindentation technology, we investigated the elastic-to-plastic transition via first pop-in event and estimated the corresponding shear stress for incipient plasticity, i.e., yielding in the three typical orientations, i.e., X-112°, Y-36°, and Y-42° planes. The occurrence of incipient plasticity exhibited a stochastic distribution in a wide range for the three orientations. Accordingly, the obtained values of yield stress were uniform and scattered in the range from about 4 to 7 GPa for LiTaO_3_ single crystal. The orientation effect on yield stress at the nano-scale was revealed to be insignificant in LiTaO_3_ single crystal. The yield stresses were 5.44 ± 0.41, 5.74 ± 0.59, and 5.34 ± 0.525 GPa for the X-112°, Y-36°, and Y-42° planes, respectively. The activation volumes of dislocation nucleation were computed based on the cumulative distribution of yield stress, which were 12 Å^3^, 8 Å^3^, and 9 Å^3^ for the X-112°, Y-36°, and Y-42° planes. The results indicated that point-like defects could be the source of plastic initiation on the surface of LiTaO_3_ single crystal.

## 1. Introduction

As a relatively new synthetic piezoelectric material lithium tantalate (LiTaO_3_) single crystal has been extensively adopted in the commercial laser and communication fields due to their prominent optical and electrical properties [1]. The most well-known application of LiTaO_3_ single crystal is in the field of surface acoustic wave (SAW) devices [2], for its low acoustic loss. The thickness and surface quality are two important factors for LiTaO_3_ single crystal, which significantly influence the efficiency and functionality of LiTaO_3_-based devices. Relying on ultra-precision thinning technology, the thickness of LiTaO_3_ single crystal can be reduced down to tens of micrometers [3]. Meanwhile, surface roughness below one nanometer can be attained. Mechanical properties and deformation mechanism on the surface of LiTaO_3_ have attracted significant attention in order to promote the thinning efficiency [4]. For an ultrathin LiTaO_3_ single crystal wafer, the risk of catastrophic brittle fracture is increased during machining processes and application service in comparison to its bulk counterpart. Tiny defects such as scratches and damage on the surface layer could result in fracture of the thinned LiTaO_3_ single crystal. Furthermore, the crystal orientation plays an important role in mechanical properties such elastic modulus, hardness, and fracture morphology in LiTaO_3_ single crystal [5,6,7]. 

Due to its hard–brittle nature, yield stress in LiTaO_3_ is difficult to measure by conventional methods. Additionally, brittle fractures of single crystal is generally governed by defects such as pores and scratches [6]. Therefore, yielding and fracture behaviors are hard to anticipate in LiTaO_3_ single crystal. Relying on nanoindentation technology, the elastic-to-plastic transition of the localized volume beneath the indenter can be accurately obtained by detecting the first pop-in event [8,9]. In recent years, the yield stress and mechanism of incipient plastic deformation under nanoindentation have attracted much attention in metallic crystals, amorphous alloys, and semiconductor materials [10,11,12]. To the authors’ best knowledge, the yielding behavior and plastic mechanism in LiTaO_3_ single crystal is not well understood. During the surface machining process, surface and/or sub-surface damage is generated. The density of defects such as dislocation and impurity is higher on the surface in comparison to the interior. Additionally, the atomic structure could be disturbed by grinding, for instance, grain size reduction and amorphization for alloys [13]. Accordingly, the incipient plastic behavior and yield stress of LiTaO_3_ single crystal at the nanoscale could be distinct to those of bulk counterpart. Furthermore, the orientation effect on yield stress at the nanoscale needs to be clarified in LiTaO_3_ single crystal. With this in mind, we aimed to reveal yielding features by nanoindentation in the three typical cleavage planes (1$\bar{1}$02), ($\bar{1}$012), and (01$\bar{1}$2) of LiTaO_3_ single crystal, i.e., the X-112°, Y-36°, and Y-42° planes.

## 2. Materials and Methods 

Prior to nanoindentation measurement, the three typical orientations of commercial LiTaO_3_ single crystal wafers were carefully polished. The final thickness was 0.25 mm and the surface roughness *R*_a_ was 1.58, 1.50, and 2.18 nm for the X-112°, Y-36°, and Y-42° planes on an area of 360 × 270 μm^2^ by optical profiler (Zygo Newview 5022), respectively [14]. A schematic illustration of the atomic arrangements of the three planes and their surface morphologies is exhibited in Supplementary Materials.

Nanoindentation tests were conducted using an Agilent Nano Indenter G200 at a constant temperature of 20 °C. A conical indenter with a diamond spherical tip was adopted, of which the nominal radius was 5 μm and its effective radius was calibrated to be 2.95 μm on standard fused silica. By using a spherical tip, the elastic deformation beneath the indenter could bear a higher load in comparison to a Berkovich indenter. Therefore, the first pop-in event, i.e., incipient plasticity, could be more clearly discerned during spherical nanoindentation. A matrix of 10 × 10 measurements was performed at each orientation with 20 μm intervals. The peak load and loading rate were 20 mN and 0.5 mN/s, respectively. The space between two adjacent nanoindentations was more than 50 times the maximum pressed depth. All the nanoindentation tests were launched until thermal drift reduced to below 0.05 nm/s. 

## 3. Results and Discussion

**Figure 1:**
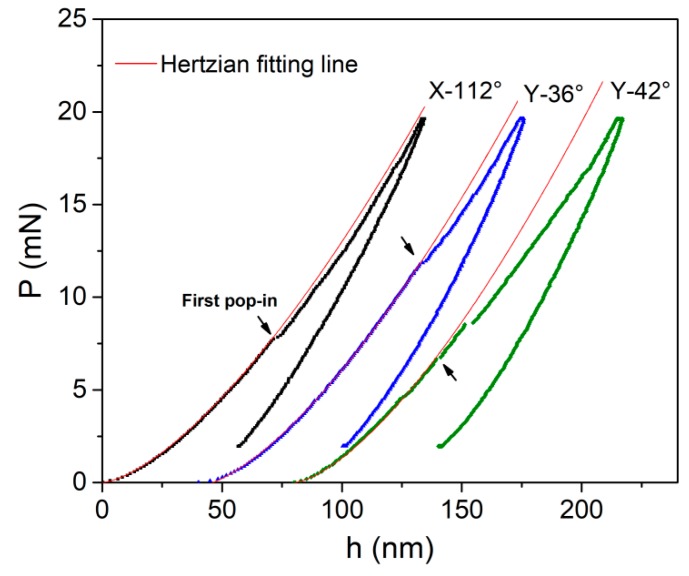
Typical load versus displacement (*P–h*) curves of X-112°, Y-36°, and Y-42° planes under spherical nanoindentation; the initial loading segment could be perfectly fitted by Hertzian contact theory. The first pop-in event currently occurred at the position of deviation point between Hertzian fitting line and loading sequence.

Figure 1 shows the typical *P–h* curves of the X-112°, Y-36°, and Y-42° planes at a maximum load of 20 mN and loading rate of 0.5 mN/s. Pop-in events with a scale of 1–3 nm were observed in each sample. The initial loading sequence can be well fitted by the Hertzian elastic contact theory [15], given by:

(1) $$P=\frac{4}{3}E_{r}\sqrt{R}h^{1.5},$$

where *E_r_* is the reduced elastic modulus which accounts for that the elastic displacement occurring in both the tip and sample. *R* is the tip radius. Elastic constant of the film can be deduced by:

(2) $$\frac{E_{s}}{1-v_{s}^{2}}={\left(\frac{1}{E_{r}}-\frac{1-v_{i}^{2}}{E_{i}}\right)}^{-1},$$

where v is the Poisson’s ratio, with the subscripts *s* and *i* representing the sample and the indenter, respectively. For the commonly used diamond tip, *E_i_* = 1141 GPa and v_I_ = 0.07. It should be mentioned that the Hertzian fitting line exactly deviated from the *P–h* curve at the position of the first pop-in in three planes. This phenomenon clearly indicated the transition from elastic to elastic–plastic deformation once the first pop-in emerges, which also could be regarded as the onset of yielding during nanoindentation. 

Based on Bei’s maximum shear stress criterion [16], the maximum shear stress *τ_m_* underneath the indenter when the first pop-in event occurred represents the shear strength for the onset of plasticity. For a spherical indenter, the *τ_m_* happens at about half the elastic contact radius $\;a=\sqrt{Rh_{e}}$ , right below the contact surface, which could be estimated by $\tau_{m}\approx 0.445P_{m}$. *P_m_* is the mean pressure beneath the indenter as the first pop-in appears, given by:

(3) $$P_{m}=\frac{P}{\pi a^{2}},$$

**Figure 2:**
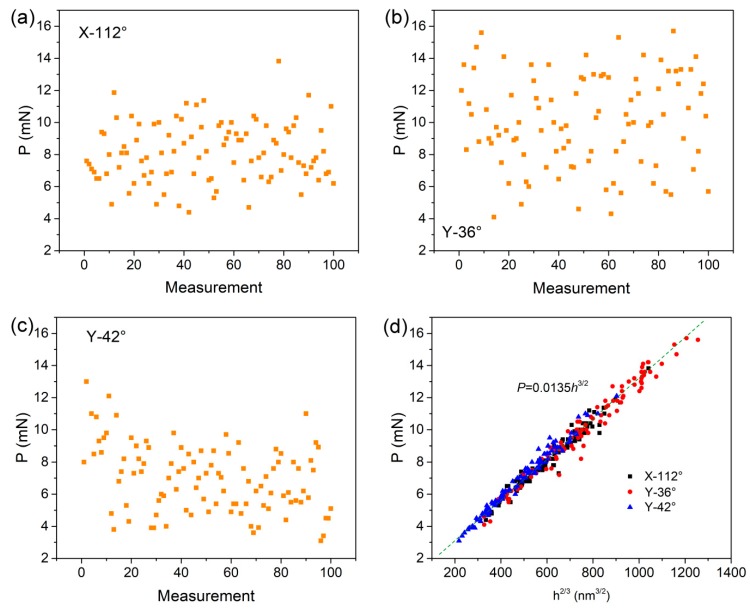
The distribution of critical load at the first pop-in event as a function of measurements for (**a**) X-112° plane, (**b**) Y-36° plane, and (**c**) Y-42° plane; (**d**) Statistics of 100 *P–h^1.5^* pairs on the first pop-in events, which followed linear correlation upon Hertzian contact theory.

*P* and *h_e_* are the critical load and displacement at first pop-in location. The critical loads at first pop-in events for three planes have been plotted with the measurements, as shown in Figure 2a–c. The occurrence of yielding was uniform and scattered under nanoindentation, probably due to the stochastic process of the dislocation nucleation. The distribution ranges of the critical load were approximately from 4 to 12 mN, 4 to 16 mN, and 3 to 11 mN for the X-112°, Y-36°, and Y-42° planes, respectively. Figure 2d shows the correlation between the critical load and displacement at first pop-in position. The power-law fitting expression is *P =* 0.0135*h*^1.5^. The almost linear distribution of 100 *P–h^1.5^* pairs in the three planes, which conformed well to the Hertzian elastic contact theory, indicated a perfect elastic deformation in LiTaO_3_ crystal. Importantly, it confirmed that the first pop-in event was linked to incipient plasticity rather than extrusion during nanoindentation. Moreover, the linear fitting lines for three planes nearly overlapped, which suggests similar elastic constants on different orientations under a spherical tip. The deduced elastic modulus on surface using Hertzian theory was 185 GPa for LiTaO_3_ single crystal. It is worth mentioning that the elastic constant found herein was much lower than previous nanoindentation results of ~220–250 GPa by Berkovich indenter at deep dislocations [5,7,14]. In spite of different testing conditions, such pronounced reduction of elastic modulus could be mainly due to structural and/or chemical change on the surface, which indicates the significant effect of the damage layer on the mechanical properties of LiTaO_3_ single crystal.

**Figure 3:**
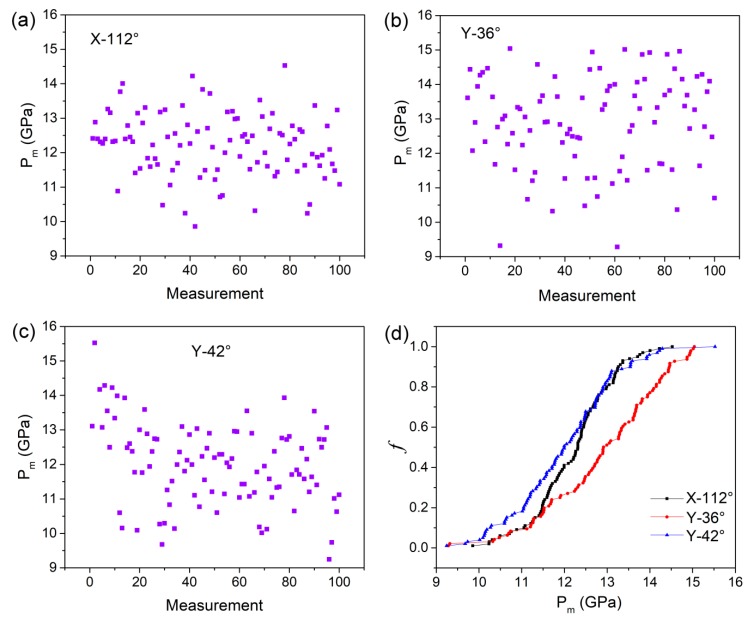
The values of maximum pressure stress *P*_m_ at the onset of first pop-in versus measurements for (**a**) X-112° plane, (**b**) Y-36° plane, and (**c**) Y-42° plane; (**d**) Cumulative distribution of *P*_m_ for the three planes.

**Figure 4:**
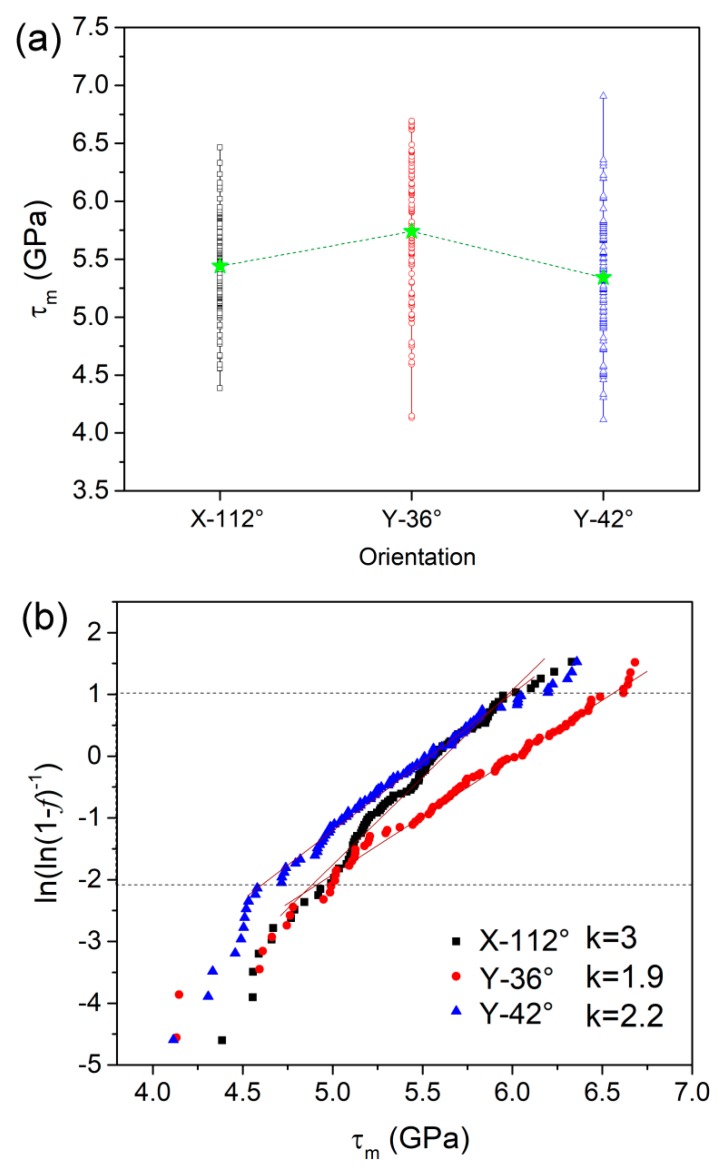
(**a**) The values of *τ*_m_ on the three orientations, in which mean value of each plane was depicted; (**b**) the correlation between ln[ln(1-*f*)^−1^] and maximum shear stress *τ*_m_ for the three planes. Linear fitting was employed to estimate the activation volume.

Figure 3a–c shows the *P_m_* values obtained from the 100 *P–h* curves as a function of measurement for the X-112°, Y-36°, and Y-42° planes, respectively. Generally, the *P_m_* values of LiTaO_3_ single crystal were also uniform and scattered in a wide range from roughly 9 to 16 GPa. Figure 3d shows the cumulative distribution of *P_m_* for the three planes. The average values of *P_m_* were 12.2, 12.9, and 12 GPa for X-112°, Y-36°, and Y-42° planes. Once the *P_m_* was determined, the yield stress and its statistical law were concomitantly obtained. The values of yield stress *τ*_m_ for three orientations are exhibited in Figure 4a, in which the mean values were 5.44, 5.74, and 5.34 GPa for the X-112°, Y-36°, and Y-42° planes, respectively. The distribution range of yield stress was 4.4 to 6.5 GPa for X-112°, 4.1 to 6.7 GPa for Y-36°, and 4.1 to 7 GPa for Y-42°. The calculated mean pressure and yield stress are listed in Table 1 for the three planes. The experiment resultal indicated orientation-independent yield stress on the surface of LiTaO_3_ single crystal. In addition, the upper limits of the yield stress for three planes were ~6.5–7 GPa, which is comparable to the ideal yield shear stress 7.8 GPa by *G*/10. The shear modulus *G* was computed by *E*/2(1 + *v*). The extremely high yield stress indicates that the incipient plasticity was triggered by dislocation nucleation in a grain [17], rather than pre-existing flaws coming from grinding or polishing. Furthermore, a uniform distribution of *P*_m_ or *τ*_m_ illustrates a homogeneous nucleation of dislocation on the surface.

According to Schuh’s original work [18], the activation volume of dislocation nucleation was estimated, which relied on the cumulative distribution of yield stress by nanoindentation. Recently, researchers have applied this statistical method to estimate the critical size for plastic initiation in new-structure materials, such as metallic glass and high-entropy alloys [19,20]. Based on Schuh’s constructions, the cumulative probability of the thermally-assisted and stress-biased shear stress *τ* can be described as:

(4) $$f=1-\exp\left[-\frac{kT{\dot{\gamma }}_{0}}{V^{*}\left(d\tau /dt\right)}\exp\left(-\frac{\Delta F^{*}}{kT}\right)\exp\left(\frac{\tau V^{*}}{kT}\right)\right],$$

where *k* is the Boltzmann constant, *T* is the temperature, ${\dot{\gamma }}_{0}$ is the attempt frequency, and $\Delta F^{*}$ is the Helmholtz activation energy, the ratio of dτ/dt is a constant in the fixed loading-rate-control mode. V* is the activation volume, which can be calculated from the slop of $\ln\left[\ln(1-f{)}^{-1}\right]$ vs. *τ* by converting the Equation (4) to be as:

(5) $$\ln\left[\ln(1-f{)}^{-1}\right]=\left\{\frac{\Delta F^{*}}{kT}+\ln\left[\frac{kT}{V^{*}\left(d\tau /dt\right)}\right]\right\}+\frac{\tau V^{*}}{kT},$$

Figure 4b shows the correlation between $\ln\left[\ln(1-f{)}^{-1}\right]$ and *τ*_m_ for all the planes. The *f* range between 10% and 90% was adopted for linear fitting, as indicated by dotted line. Accordingly, the activation volume of dislocation nucleation in LiTaO_3_ single crystal could be obtained: respectively, 12, 8, and 9 Å^3^ on the X-112°, Y-36°, and Y-42° orientations. The estimated activation volumes of dislocation nucleation in LiTaO_3_ single crystal were consistent with previous reported ranges in single and poly-crystalline metals [21,22]. The activation volumes of the three planes were at the magnitude of single atomic volume, which seemed independent of crystal orientation. Point-like defects such as vacancies and impurities could be assumed to be the sources of dislocation nucleation. It is reasonable that these defects were easily generated on the surface of LiTaO_3_ by polishing. The activation volume of plasticity unit could also be tightly tied to the ductility of materials [23,24]. However, for the hard–brittle LiTaO_3_ single crystal, more investigation is required to illustrate the intrinsic correlation between the activation volume of dislocation nucleation and deformation characteristic.

From the perspective of atomic arrangement, amorphous structures such as metallic glass have lower elastic constants and higher yield stress (due to larger elastic limit), in comparison to crystalline structure with the same composition. It could be assumed that crystal arrangement on the surface was severely agitated by polishing, i.e., non-crystallization of the surface structure. Therefore, the low elastic modulus and nearly ideal yield stress could be qualitatively explained. Accordingly, the crystal orientation effect on mechanical properties no longer existed. Intrinsically, similar values of activation volumes of dislocation nucleation suggested the approximate activation energies for incipient plasticity on the three planes. As a consequence, the low difference of yield stress among three planes could be explained based on plastic mechanism. Investigation of the damage layer structure on the surface was outside the scope of this work, and needs further high-resolution observation of atomic structure in detail.

## 4. Conclusions

Relying on spherical nanoindentation, yield stresses at the nano-scale in LiTaO_3_ single crystal were estimated. The orientation effect on yield stress was weak, and the mean values were 5.44 ± 0.41, 5.74 ± 0.59, and 5.34 ± 0.525 GPa for the X-112°, Y-36°, and Y-42° planes. Based on the statistical law of yield stress, activation volumes of dislocation nucleation were computed as 12 Å^3^, 8 Å^3^, and 9 Å^3^ for the X-112°, Y-36°, and Y-42° planes, respectively. It was indicated that point-like defects could be the main sources of plastic initiation on the surface of LiTaO_3_ single crystal.

## Figures and Tables

**Table 1:** Mean pressure, yield stress, and activation volume for three planes.

Orientation	Mean Pressure *P*_m_, GPa	Yield Stress *τ*_m_, GPa	Activation Volume, Å^3^
X-112°	12.2 ± 0.92	5.44 ± 0.41	12
Y-36°	12.9 ± 1.33	5.74 ± 0.59	8
Y-42°	12 ± 1.18	5.34 ± 0.525	9

## Supplementary Materials

The following are available online at https://www.mdpi.com/1996-1944/12/17/2799/s1, Figure S1: Schematic illustration of atomic arrangements for the typical orientations (a) X-112°, (b) Y-36°, and (c) Y-42° in LiTaO3 single crystal, and their surface morphologies by optical profile.
