# Supplementary material for: Orientation-Independent Yield Stress and Activation Volume of Dislocation Nucleation in LiTaO3 Single Crystal by Nanoindentation
Source: Materials (Basel). 2019 Aug 30;12(17):2799. doi: 10.3390/ma12172799 (PMC6747799; doi:10.3390/ma12172799)
Supplement: Supplementary file 1 [file materials-12-02799-s001.pdf]

materials

Supplementary Materials

Orientation-Independent Yield Stress and Activation Volume of Dislocation Nucleation in $LiTaO_3$ Single Crystal by Nanoindentation

Yi Ma [1], Xianwei Huang [1], Yuxuan Song [1], Wei Hang [1,2,*], Julong Yuan [1,2] and Taihua Zhang [3,*]

1 College of Mechanical Engineering, Zhejiang University of Technology, Hangzhou 310014, China
2 Key Laboratory of Special Purpose Equipment and Advanced Manufacturing Technology Ministry of Education, Zhejiang University of Technology, Hangzhou 310027, China
3 Institute of Solid Mechanics, Beihang University, Beijing 100191, China
* Correspondence: whang@zjut.edu.cn (H.W.); zhangth66@buaa.edu.cn (Z.T.H.)

$LiTaO_3$ single crystal is belonging to trigonal *R*3*c* space group with ion bonding structure and commonly depicted by hexagonal axes. Figure S1 shows the schematic illustration of atomic arrangement in $LiTaO_3$ single crystal and the typical orientations $(1\bar{1}02)$, $(\bar{1}012)$ and $(01\bar{1}2)$ i.e., X-112°, Y-36° and Y-42° were displayed in Fig. 1(a)-(c), respectively. Li atom lies in an oxygen layer that is c/4 away from the Ta atom, and Ta atom is centered between oxygen layers. The representative surface morphologies of three planes were also exhibited in Figure S1 below the lattice diagrams.

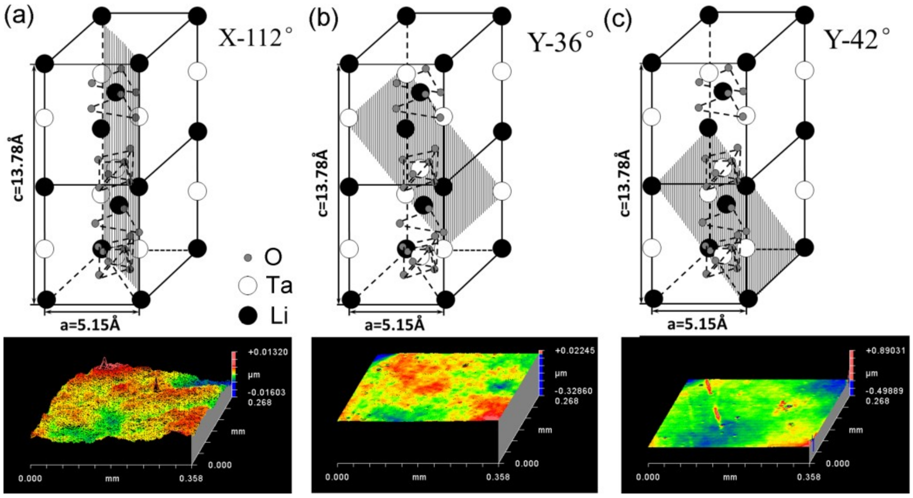

Figure S1. Schematic illustration of atomic arrangements for the typical orientations (a) X-112°, (b) Y-36° and (c) Y-42° in $LiTaO_3$ single crystal, and their surface morphologies by optical profile. See *Ma, Y.; Huang, X.W.; Song, Y.X. et al. Materials* 2019, *12*, *1683*.
